# Protein Palmitoylation as a Molecular Switch Linking Regulated Cell Death and Disease

**DOI:** 10.3390/biom16060853

**Published:** 2026-06-11

**Authors:** Xiaozhe Liu, Likun Cheng, Mingcheng Liu, Mingzhu Zhou, Bingze Jiao, Xuehan Liu, Jianhe Hu, Yanwei Li, Xiaojing Xia

**Affiliations:** 1 College of Animal Science and Veterinary Medicine, Henan Institute of Science and Technology, Xinxiang 453003, China; lxz@stu.hist.edu.cn (X.L.); liumc@hist.edu.cn (M.L.); 1552109252@stu.hist.edu.cn (M.Z.); jbz@stu.hist.edu.cn (B.J.); duanmingyuan@stu.hist.edu.cn (X.L.); hujianhe@hist.edu.cn (J.H.); 2 Shandong Binzhou Animal Science and Veterinary Medicine Academy, Research Institution of Veterinarian, Binzhou 256600, China; jianghai730@gmail.com

**Keywords:** protein palmitoylation, S-acylation, regulated cell death, cell death crosstalk, cancer, neurodegeneration, inflammatory signaling, therapeutic targets

## Abstract

Regulated cell death is essential for tissue homeostasis, immune defense, and disease progression, yet the lipid-based regulatory mechanisms that coordinate cell death signaling remain incompletely understood. Protein palmitoylation is a dynamic and reversible lipid post-translational modification that controls protein membrane association, trafficking, stability, and signaling complex assembly. This review summarizes the regulatory roles of palmitoylation and depalmitoylation in major forms of regulated cell death, including apoptosis, necroptosis, pyroptosis, ferroptosis, and autophagy-related cell death. Particular attention is given to representative palmitoylated substrates, including Fas cell surface death receptor (Fas), receptor-interacting protein kinase 1 (RIPK1), NLR family pyrin domain containing 3 (NLRP3), gasdermin D (GSDMD), glutathione peroxidase 4 (GPX4), solute carrier family 7 member 11 (SLC7A11), autophagy-related 16 like 1 (ATG16L1), and Beclin1. These substrates illustrate how palmitoylation links membrane organization, metabolic status, inflammatory signaling, and cell fate decisions. Disease-oriented evidence further indicates that dysregulated palmitoylation contributes to cancer, neurodegenerative diseases, and inflammatory or immune-related disorders by modulating cell death resistance, inflammatory amplification, immune evasion, or impaired proteostasis. Current challenges include limited quantitative information on palmitoylation dynamics, incomplete evidence for some enzyme–substrate relationships, and insufficient distinction between disease-driving and secondary palmitoylation events. Targeting zinc finger Asp-His-His-Cys (zDHHC) palmitoyl acyltransferases, depalmitoylating enzymes, or specific palmitoylated substrates may provide new therapeutic opportunities. Overall, this review positions protein palmitoylation as a dynamic molecular switch linking lipid metabolism, membrane signaling, regulated cell death, and disease remodeling.

## 1. Introduction

Cell death is a fundamental biological process essential for maintaining tissue homeostasis, developmental progression, and immune defense in multicellular organisms [1]. In recent years, the identification and characterization of multiple forms of programmed cell death, including apoptosis, necroptosis, pyroptosis, ferroptosis, and autophagy-related cell death, have placed cell fate regulation at the forefront of physiological and pathological research [2]. Increasing evidence indicates that, beyond classical transcriptional regulation and protein phosphorylation, lipid post-translational modifications also play critical roles in cell death signaling networks, among which protein palmitoylation has attracted particular attention [3]. As a dynamic and reversible lipid modification, palmitoylation not only regulates protein membrane localization and stability but also participates in signaling complex assembly, inflammatory amplification, and cell death pathway selection, emerging as a key molecular hub linking metabolic status, cell death, and disease phenotypes [4].

Protein palmitoylation is one of the most prevalent lipid post-translational modifications and provides a structural basis for membrane targeting and signal regulation [5,6]. Among different forms of palmitoylation, S-palmitoylation is the most biologically significant because palmitate is attached to cysteine residues through a reversible thioester bond [6]. This reversibility allows substrate proteins to cycle between palmitoylated and depalmitoylated states in response to cellular signals and microenvironmental changes [7]. Mechanistically, zinc finger Asp-His-His-Cys (zDHHC) family palmitoyl acyltransferases catalyze the transfer of palmitoyl groups from palmitoyl-CoA to substrate proteins, whereas acyl protein thioesterases (APTs), palmitoyl-protein thioesterases (PPTs), and other depalmitoylating enzymes reverse this modification [8,9,10]. Through this dynamic cycle, palmitoylation regulates protein hydrophobicity, membrane localization, vesicular trafficking, stability, conformational state, and protein–protein interactions, thereby influencing signaling complex assembly and cellular response thresholds [5,11]. Recent studies further suggest that palmitoylation functions as an important molecular interface linking metabolic status, inflammatory signaling, and regulated cell death pathways [12,13].

Several recent reviews have systematically summarized the role of palmitoylation in regulated cell death, including apoptosis, necroptosis, pyroptosis, ferroptosis, autophagy-related cell death, and other emerging forms of cell death [12,13]. These studies have provided valuable overviews of palmitoylated substrates, regulatory enzymes, pathway-specific mechanisms, and therapeutic implications. Nevertheless, several important issues remain insufficiently integrated. The strength of evidence supporting individual palmitoylation events varies considerably across different pathways, ranging from directly validated modification sites and enzyme–substrate relationships to indirect or context-dependent associations. Moreover, whether a given palmitoylation event functions as a disease-driving mechanism or represents a secondary adaptive response is often unclear. The determinants governing whether palmitoylation promotes cell death or supports cytoprotective signaling also remain incompletely understood. These unresolved issues are particularly important in disease settings, where the same palmitoylation event may produce distinct biological outcomes depending on substrate identity, enzyme selectivity, cell type, metabolic status, and pathological context.

Therefore, this review provides an updated and disease-oriented synthesis of current knowledge regarding palmitoylation-mediated regulation of regulated cell death. After outlining the fundamental mechanisms of palmitoylation and depalmitoylation, this review focuses on the key regulatory nodes through which palmitoylation modulates apoptosis, necroptosis, pyroptosis, ferroptosis, and autophagy-related cell death. It further discusses the integrated roles of palmitoylation in the crosstalk among distinct cell death pathways, inflammatory amplification, metabolic stress responses, and disease progression. Compared with existing reviews, this article places greater emphasis on the spatiotemporal specificity of palmitoylation, enzyme–substrate selectivity, and its context-dependent bidirectional effects in either promoting cell death or supporting cytoprotective signaling. By highlighting its pathological relevance and potential therapeutic value in cancer, neurodegenerative diseases, and inflammatory/metabolic disorders, this review provides a framework for understanding palmitoylation-mediated regulated cell death networks and for developing targeted intervention strategies.

## 2. Literature Search Strategy

This review focuses on the role of protein palmitoylation in the regulation of cell death and disease-associated mechanisms. To improve the completeness of literature coverage and the transparency of the review process, relevant English-language publications published from January 2006 to March 2026 were searched and integrated from PubMed, Web of Science, and Scopus. Considering the rapid development of this field in recent years, priority was given to recent publications, especially those published from 2022 to 2026, which accounted for the majority of the included references. A small number of earlier or foundational studies were retained when they provided essential mechanistic background on regulated cell death, protein palmitoylation, zDHHC enzymes, depalmitoylation, or palmitoylation detection methods. The search terms included “protein palmitoylation,” “S-palmitoylation,” “zDHHC,” “depalmitoylation,” “regulated cell death,” “apoptosis,” “necroptosis,” “pyroptosis,” “ferroptosis,” “autophagy-related cell death,” “cell death crosstalk,” “inflammation,” “cancer,” “neurodegeneration,” and “therapeutic targeting.” Boolean operators, including “AND” and “OR,” were used to combine search terms where appropriate.

During literature screening, priority was given to the following categories of studies: (1) original research directly reporting the regulation of cell death-related molecules by protein palmitoylation or depalmitoylation; (2) mechanistic studies that clearly identified palmitoylation sites, regulatory enzymes, cellular or animal models, and functional validation evidence; (3) studies addressing palmitoylation-mediated regulation of cell death in cancer, neurodegenerative diseases, inflammatory diseases, or metabolic disorders; and (4) representative reviews and classic studies closely related to the central theme of this article.

The exclusion criteria included (1) duplicate records; (2) conference abstracts, editorials, commentaries, and non-peer-reviewed publications; (3) studies lacking experimental evidence for palmitoylation-related mechanisms; (4) studies based solely on bioinformatic prediction without functional validation; and (5) publications not directly relevant to protein palmitoylation, regulated cell death, or disease-associated mechanisms. Articles meeting the inclusion criteria were further evaluated and integrated according to their mechanistic relevance, experimental evidence, and contribution to the understanding of palmitoylation-mediated regulation of cell death.

The literature-selection process followed a PRISMA-style workflow. A total of 1286 records were initially identified from the three databases. After removal of 342 duplicate records, 944 records remained for title and abstract screening. Subsequently, 216 full-text articles were assessed for eligibility, of which 129 articles were excluded according to the predefined criteria. Ultimately, 87 studies were included in the qualitative synthesis. The detailed literature-selection process is summarized in Supplementary Figure S1.

## 3. Molecular Basis and Dynamic Regulation of Protein Palmitoylation

### 3.1. Chemical Basis and Dynamic Features of Palmitoylation

Palmitoylation is a lipid post-translational modification in which a 16-carbon saturated fatty acid, palmitate, is covalently attached to specific sites on substrate proteins. It mainly includes S-palmitoylation and N-palmitoylation, among which S-palmitoylation—linked via a thioester bond to cysteine residues—is the most common and biologically significant form [14]. Unlike relatively stable lipid modifications such as myristoylation and prenylation, S-palmitoylation is highly reversible, enabling dynamic regulation of protein membrane association, dissociation, and signaling responses. The core enzymes responsible for this process are the zDHHC family of palmitoyl acyltransferases (PATs), which contain a conserved Asp-His-His-Cys (DHHC) catalytic motif and utilize palmitoyl-CoA as the acyl donor to transfer palmitate to substrate proteins [15,16]. The catalytic process typically involves formation of an enzyme–palmitoyl intermediate, followed by transfer of the palmitoyl group to the target protein. This process is influenced by local lipid availability, membrane environment, pH, and redox status, highlighting palmitoylation as a metabolically sensitive and spatially regulated dynamic modification [17].

To date, 23 zDHHC family members have been identified in mammals and are distributed across the Golgi apparatus, endoplasmic reticulum, plasma membrane, and other membrane compartments, exhibiting distinct substrate preferences [18]. Structurally, zDHHC enzymes are polytopic membrane proteins that are generally predicted to contain four transmembrane domains and a conserved cysteine-rich Asp-His-His-Cys (DHHC) catalytic domain located on the cytosolic face of the membrane. Structural studies of zDHHC20 and zDHHC15 revealed a tepee-like transmembrane architecture that forms a hydrophobic cavity for acyl-CoA binding and positions the catalytic cysteine at the membrane–cytosol interface, providing the structural basis for autoacylation and subsequent acyl transfer [4,19,20]. In addition to the catalytic domain, several zDHHC proteins contain accessory regions that contribute to substrate recruitment and specificity. For example, zDHHC17 and zDHHC13 possess N-terminal ankyrin repeat domains that recognize specific short linear motifs in selected substrates and thereby facilitate substrate recruitment and selective palmitoylation [21,22]. Computational tools such as CSS-Palm have also been developed to predict potential palmitoylation sites in substrate proteins; however, these predictions do not directly define enzyme–substrate specificity and require experimental validation [23]. These structural and functional features help explain why selective targeting of individual zDHHC enzymes remains challenging because of substrate overlap, membrane localization constraints, and context-dependent substrate availability.

### 3.2. The Depalmitoylation System and Its Regulatory Roles

**Figure 1:**
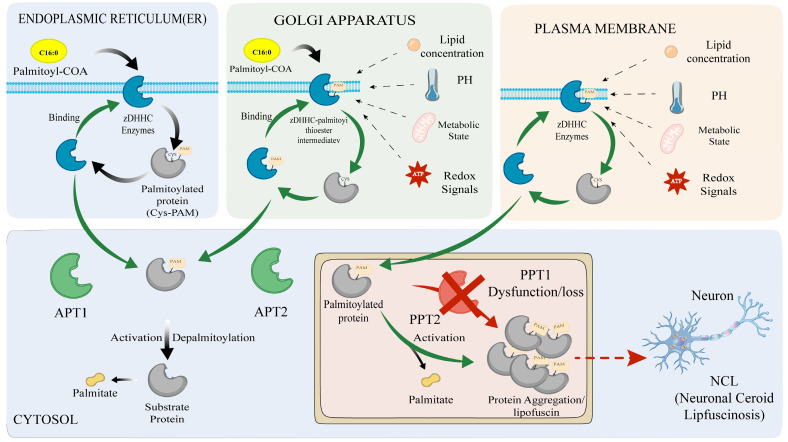
Dynamic cycle of protein palmitoylation and depalmitoylation. Protein palmitoylation is catalyzed by zDHHC palmitoyl acyltransferases using palmitoyl-CoA as the acyl donor, whereas depalmitoylation is mediated by thioesterases such as APT1/2 and PPT1/2. This reversible cycle dynamically regulates substrate membrane association and subcellular trafficking among the endoplasmic reticulum, Golgi apparatus, plasma membrane, cytosol, and lysosome, as well as protein stability, turnover, and signaling activity. Disruption of this balance, such as impaired lysosomal depalmitoylation caused by PPT1 dysfunction, may lead to the accumulation of palmitoylated proteins and contribute to neuronal ceroid lipofuscinosis. Colors distinguish molecule types: yellow, palmitoyl-CoA/palmitate; blue, zDHHC enzymes; green, depalmitoylating enzymes; gray, substrate proteins. Black/green arrows indicate palmitoylation–depalmitoylation cycling or trafficking, while the red cross and dashed red arrow indicate PPT1 dysfunction and its link to NCL.

In contrast to palmitoylation, depalmitoylation is a key process that maintains the dynamic balance of this modification and is primarily mediated by depalmitoylating enzymes such as palmitoyl protein thioesterase 1 and 2 (PPT1 and PPT2), acyl protein thioesterases 1 and 2 (APT1 and APT2), and related α/β-hydrolase domain-containing proteins such as ABHD17 family members [24]. These enzymes are distributed in the cytosol, lysosomes, and associated membrane compartments, where they cleave the thioester bond between palmitate and substrate proteins, converting them from a membrane-bound to a soluble state and enabling relocalization or functional remodeling. Different depalmitoylating enzymes have distinct cellular roles: APT1/2 mainly mediate rapid cycling between cytosol and membranes, thereby regulating short-term signaling events such as receptor clustering, kinase activation, mitochondrial targeting, and inflammatory signaling; in contrast, PPT1/2 are more closely associated with lysosomal protein turnover and long-term homeostasis [25,26]. For example, APT1-dependent depalmitoylation participates in the spatial cycling of palmitoylated RAS proteins, whereas ABHD17A-mediated depalmitoylation limits NLRP3 inflammasome activation. Dysregulation of lysosomal depalmitoylation can also lead to pathological consequences; PPT1 mutations cause the accumulation of palmitoylated proteins in neurons, forming lipofuscin-like deposits and contributing to neuronal ceroid lipofuscinosis (NCL) [27,28]. Thus, depalmitoylation is not merely a removal process but a critical regulatory mechanism for signal termination, protein turnover, cellular homeostasis, and regulated cell death signaling. Together, palmitoylation and depalmitoylation constitute a dynamic and reversible lipid modification system that regulates substrate membrane association, trafficking between membrane compartments and the cytosol, protein turnover, and signaling activity across distinct subcellular compartments (Figure 1).

### 3.3. Biological Effects of Palmitoylation on Protein Function

The biological significance of palmitoylation extends beyond lipid attachment itself, as this modification reshapes substrate protein function through three interconnected mechanisms: spatial localization, protein stability, and trafficking-dependent signaling organization. First, palmitoylation increases local hydrophobicity and promotes membrane association or enrichment within specialized membrane microdomains, such as lipid rafts, thereby facilitating receptor clustering and signaling complex assembly. For example, palmitoylation of the Fas receptor enhances its localization to lipid rafts and promotes death-inducing signaling complex formation [29]. Second, palmitoylation regulates protein stability and turnover by influencing ubiquitination-dependent degradation, lysosomal trafficking, or cell-surface retention. A representative example is programmed death-ligand 1 (PD-L1), whose Cys272 palmitoylation stabilizes PD-L1, reduces its degradation, and promotes tumor immune evasion [30,31]. Third, palmitoylation controls intracellular trafficking and subcellular distribution by regulating the movement or retention of proteins among the Golgi apparatus, endosomes, lysosomes, mitochondria, and plasma membrane. For instance, AKT palmitoylation promotes membrane anchoring and activation in high-fat diet-associated liver tumorigenesis, whereas zDHHC7-mediated palmitoylation of ATG16L1 facilitates membrane recruitment, LC3 lipidation, and autophagosome formation [32,33]. Collectively, these mechanisms illustrate that palmitoylation functions not merely as a membrane-targeting modification but as a dynamic organizer of signaling networks that integrates membrane compartmentalization, protein turnover, and intracellular trafficking, thereby influencing cellular responses including survival, inflammation, autophagy, and regulated cell death.

## 4. Palmitoylation in Regulated Cell Death Pathways

The biological functions of palmitoylation extend beyond maintaining membrane protein localization, as it broadly participates in multiple forms of programmed cell death (PCD) by regulating the membrane anchoring, complex assembly, and activation status of key signaling molecules [18]. Current evidence indicates that palmitoylation influences receptor clustering, mitochondrial membrane permeability, membrane pore formation, and inflammatory signal amplification across different cell death pathways, thereby determining the cellular response threshold to death stimuli and subsequent downstream effects [12]. Given the extensive crosstalk among apoptosis, necroptosis, pyroptosis, ferroptosis, and autophagy-related cell death, palmitoylation serves as an important regulatory layer integrating these complex networks [30]. Representative palmitoylated proteins and their functional consequences in major regulated cell death pathways are summarized in Table 1.

### 4.1. Palmitoylation and Apoptosis

**Figure 2:**
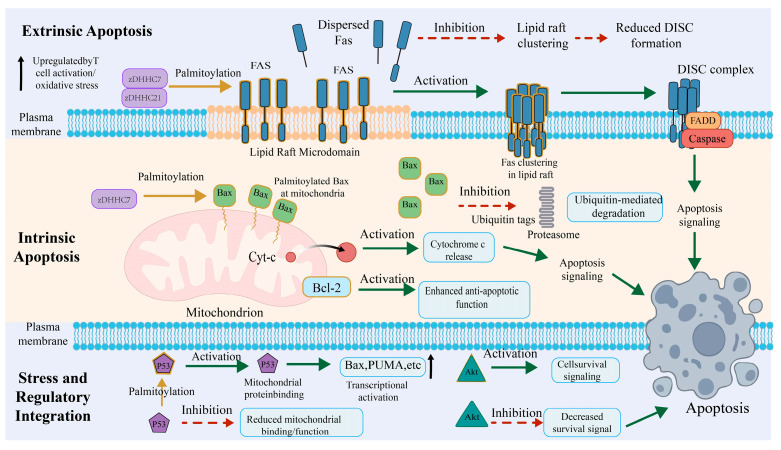
**Palmitoylation regulates apoptotic signaling through death receptor clustering, mitochondrial pathway modulation, and stress-responsive transcriptional control.** Palmitoylation promotes the membrane localization and lipid raft enrichment of death receptors such as Fas, facilitating the recruitment of FADD and Caspase-8 and subsequent formation of the death-inducing signaling complex (DISC). In the intrinsic apoptotic pathway, palmitoylation may regulate mitochondrial targeting and stability of Bcl-2 family proteins, including Bax and Bcl-2, thereby influencing mitochondrial outer membrane permeabilization, cytochrome c release, and downstream caspase activation. Palmitoylation may also modulate stress-responsive regulators such as p53 by affecting its subcellular localization and transcriptional activity toward pro-apoptotic genes such as *Bax* and *PUMA*. These effects are substrate- and context-dependent, indicating that palmitoylation can either enhance or restrain apoptotic signaling. Purple shapes indicate zDHHC enzymes, green shapes indicate apoptosis-related regulatory proteins, blue membrane regions indicate lipid raft microdomains, and cyan boxes indicate regulatory processes or signaling outputs. Orange arrows represent palmitoylation events, green arrows indicate activation or positive regulation, and red dashed arrows indicate inhibitory or attenuating effects.

Apoptosis is the most well-characterized form of programmed cell death, characterized by cell shrinkage, nuclear condensation, DNA fragmentation, and apoptotic body formation [34]. Based on activation mechanisms, apoptosis is broadly classified into the extrinsic death receptor pathway and the intrinsic mitochondrial pathway, both of which contain membrane-associated or organelle-associated regulatory nodes influenced by palmitoylation (Figure 2). In extrinsic apoptosis, death receptors such as Fas, tumor necrosis factor receptor (TNFR), and TNF-related apoptosis-inducing ligand receptor (TRAIL-R) require clustering within membrane microdomains for efficient signal transduction, with the Fas/FasL axis being one of the most extensively studied models [35]. Fas undergoes reversible palmitoylation at cysteine residues in its juxtamembrane region, primarily mediated by zDHHC7 in the currently available mechanistic evidence, which promotes its enrichment in lipid rafts and enhances recruitment of Fas-associated death domain protein (FADD) and Caspase-8 to form the death-inducing signaling complex (DISC). Inhibition of zDHHC7 activity or reduction of Fas palmitoylation markedly impairs DISC assembly and attenuates extrinsic apoptotic signaling [29,36,37]. In intrinsic apoptosis, mitochondrial outer membrane permeabilization (MOMP) is tightly regulated by the Bcl-2 family. Palmitoylation of the pro-apoptotic protein Bax at Cys126 promotes its insertion into the mitochondrial outer membrane and facilitates cytochrome c release, thereby triggering downstream caspase activation. In contrast, reduced Bax palmitoylation impairs its mitochondrial localization and attenuates apoptotic signaling [38,39]. Meanwhile, the membrane anchoring stability of the anti-apoptotic protein Bcl-2 is also regulated by palmitoylation, which enhances its retention on the mitochondrial membrane and thereby strengthens its anti-apoptotic function [40].

Beyond directly targeting death receptors and mitochondrial effectors, palmitoylation also influences apoptosis by regulating stress-responsive transcription factors and survival signaling pathways. For example, under oxidative stress or DNA damage, palmitoylation of p53 may affect its subcellular localization and transcriptional regulation of pro-apoptotic genes such as Bax and PUMA, whereas palmitoylation of survival signaling molecules like Akt helps maintain their membrane localization and anti-apoptotic signaling output [39,41,42]. Overall, palmitoylation exhibits a clear bidirectional regulatory role in apoptosis: it can enhance apoptotic signaling by promoting death receptor clustering and Bax mitochondrial targeting, while also suppressing cell death by stabilizing proteins such as Bcl-2 and Akt, thereby finely controlling cell fate decisions through modulation of membrane localization, complex assembly, and signal integration.

### 4.2. Palmitoylation and Necroptosis

**Figure 3:**
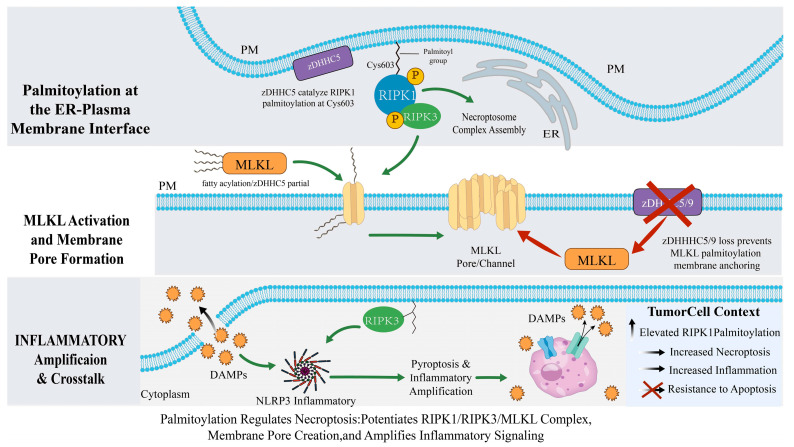
Palmitoylation controls necroptosis by regulating RIPK1 activation, necrosome assembly, MLKL membrane execution, and inflammatory amplification. Palmitoylation of RIPK1 facilitates its membrane-associated localization and promotes RIPK1–RIPK3 necrosome formation. Activated RIPK3 phosphorylates MLKL, which subsequently translocates to the plasma membrane to execute necroptotic membrane disruption. Palmitoylation-related membrane anchoring may support MLKL membrane association and necroptotic execution, although the precise site- and enzyme-specific mechanisms require further clarification. Necroptosis-associated membrane rupture promotes the release of damage-associated molecular patterns (DAMPs), thereby amplifying inflammatory signaling and potential crosstalk with inflammasome-related pathways. Purple shapes indicate zDHHC5/9 enzymes, blue and green shapes indicate RIPK1/RIPK3 necroptosis components, orange shapes indicate MLKL, yellow structures indicate MLKL-associated membrane pores, and circular particles indicate released DAMPs. Green or cyan arrows indicate activation, palmitoylation, or complex assembly, black arrows indicate DAMP release, red arrows indicate MLKL-mediated membrane disruption, and red crosses indicate inhibition or loss of zDHHC5/9-mediated palmitoylation.

Necroptosis is a form of programmed non-apoptotic cell death that depends on the RIPK1–RIPK3–MLKL (mixed lineage kinase domain-like) signaling axis and is characterized by cell swelling, plasma membrane rupture, and the release of inflammatory mediators [43]. Current evidence indicates that palmitoylation in necroptosis primarily regulates signaling complex assembly, RIPK1 membrane-associated activation, MLKL membrane execution, and inflammatory amplification (Figure 3). As a central upstream regulator, RIPK1 can undergo palmitoylation at Cys603, which promotes its enrichment at plasma membrane–endoplasmic reticulum contact sites and facilitates the formation of the RIPK1–RIPK3 complex, known as the necrosome [44]. Inhibition of the depalmitoylating enzyme APT2 enhances RIPK1 autophosphorylation and sustains its activation, thereby promoting downstream MLKL activation and necroptosis. In certain tumor contexts, elevated levels of RIPK1 palmitoylation maintain persistent signaling activity, which not only amplifies inflammatory responses but also interferes with canonical apoptotic pathways, contributing to therapy resistance [45]. These findings suggest that RIPK1 palmitoylation not only regulates necroptosis initiation but may also influence the switching between different cell death modalities.

As the key executioner of necroptosis, MLKL is phosphorylated by RIPK3, translocates to the plasma membrane, and inserts into it, ultimately disrupting membrane integrity. Studies have shown that the N-terminal region of MLKL contains multiple potential palmitoylation sites (e.g., Cys18, Cys24, and Cys32), and modification at these sites enhances its interaction with membrane phospholipids and promotes stable membrane anchoring [46]. Under conditions where zDHHC5 or zDHHC9 expression is reduced, MLKL membrane localization is impaired, leading to attenuated necroptosis. Meanwhile, necroptosis is often accompanied by pronounced inflammatory output; the released damage-associated molecular patterns (DAMPs) can further activate surrounding immune cells and inflammasome signaling, thereby amplifying the response [47]. Emerging evidence suggests that palmitoylation of RIPK3 may also enhance its interaction with NLR family pyrin domain-containing 3 (NLRP3), promoting inflammasome activation and enabling crosstalk between necroptosis and pyroptosis at the molecular level [48]. Overall, palmitoylation regulates necroptosis primarily by controlling RIPK1 complex assembly, MLKL membrane anchoring, and the coupling between necroptosis and inflammatory signaling, thereby acting as a key molecular node in inflammatory cell death.

### 4.3. Palmitoylation and Pyroptosis

**Figure 4:**
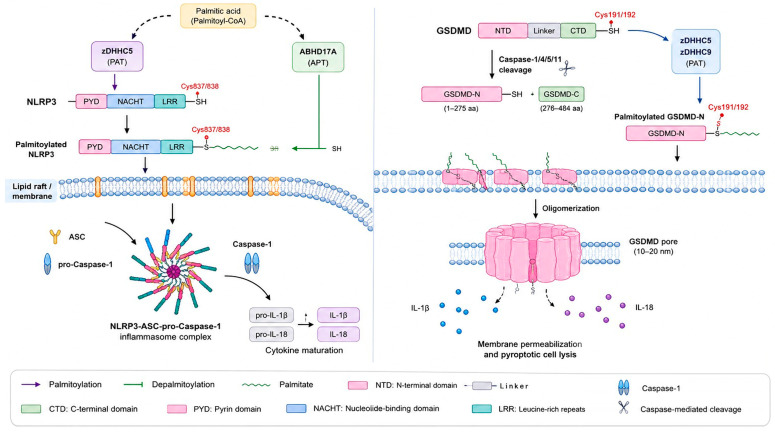
Palmitoylation promotes pyroptosis by coordinating inflammasome activation and gasdermin-mediated pore formation. zDHHC5-mediated palmitoylation of NLRP3 at Cys837/Cys838 promotes its membrane-associated localization and facilitates NLRP3–NEK7-dependent inflammasome assembly, whereas ABHD17A-mediated depalmitoylation limits NLRP3 activation. In the execution phase, zDHHC5/zDHHC9-mediated palmitoylation of GSDMD at Cys191/192 promotes GSDMD-N membrane targeting, oligomerization, pore formation, IL-1β/IL-18 release, and pyroptotic cell lysis. Purple shapes indicate zDHHC5/9 enzymes, yellow shapes indicate ABHD17A-mediated depalmitoylation, pink shapes indicate GSDMD domains or fragments, orange regions indicate lipid raft microdomains, and cyan/blue components indicate NLRP3 domains, membrane structures, or caspase-related signaling components. Green arrows indicate palmitoylation/depalmitoylation events, purple arrows indicate inflammasome or pyroptosis activation, dotted arrows indicate regulatory influence, and black arrows indicate pore formation or IL-1β/IL-18 release.

Pyroptosis is a prototypical form of inflammatory programmed cell death mediated primarily by the Gasdermin family, characterized by membrane pore formation, release of intracellular contents, and secretion of pro-inflammatory cytokines [49]. In canonical pyroptosis, GSDMD is cleaved by Caspase-1/4/5/11 to release an N-terminal active fragment that inserts into the plasma membrane to form pores, ultimately leading to cell lysis and inflammatory amplification [2]. Emerging evidence indicates that palmitoylation participates in pyroptosis both by regulating upstream inflammasome assembly and downstream gasdermin-mediated membrane pore formation (Figure 4). The NLRP3 inflammasome serves as a key platform for pyroptosis initiation, and its activation depends on precise spatial localization and complex assembly. zDHHC5-mediated palmitoylation of NLRP3 at Cys837/Cys838 promotes its interaction with NEK7 and facilitates inflammasome assembly, whereas ABHD17A-mediated depalmitoylation limits NLRP3 activation. These findings indicate that reversible NLRP3 palmitoylation functions as a spatial regulatory mechanism controlling inflammasome assembly, IL-1β maturation, and pyroptotic inflammatory amplification [16,50].

As the core executioner of pyroptosis, activation of GSDMD depends not only on caspase-mediated cleavage but also on subsequent regulation of membrane anchoring and pore formation. Palmitoylation of GSDMD at Cys191/192, mediated by zDHHC5 and zDHHC9, promotes GSDMD oligomerization, membrane pore formation, and pyroptotic execution [18,51]. Beyond directly regulating NLRP3 and GSDMD, palmitoylation may contribute to the metabolic regulation of pyroptosis. Altered lipid availability may influence pyroptosis-related palmitoylation in specific cellular and disease contexts, potentially affecting inflammasome activation and inflammatory outputs [52]. Overall, palmitoylation regulates pyroptosis primarily by controlling NLRP3 inflammasome assembly, GSDMD pore formation, and the coupling between metabolic status and inflammatory sensitivity, making it a key molecular node in pyroptosis and its disease consequences.

### 4.4. Palmitoylation and Ferroptosis

**Figure 5:**
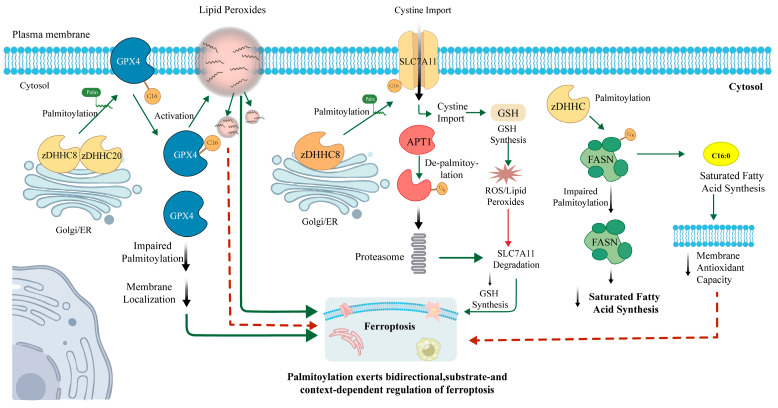
Palmitoylation regulates ferroptosis by modulating antioxidant defense, cystine transport, and lipid metabolic remodeling. Palmitoylation of GPX4, mediated by zDHHC8 or zDHHC20 and reversed by APT2, supports GPX4 stability and anti-ferroptotic function. zDHHC8-mediated palmitoylation of SLC7A11 promotes cystine uptake and glutathione synthesis, thereby enhancing resistance to lipid peroxidation. Palmitoylation-related regulation of lipid metabolic enzymes may further reshape membrane lipid composition and influence cellular susceptibility to ferroptotic stress. Yellow/orange shapes indicate zDHHC8/20 palmitoyl acyltransferases or palmitoylation-related components, red shapes indicate APT-mediated depalmitoylation, blue shapes indicate GPX4, orange membrane proteins indicate SLC7A11, and green clustered structures indicate FASN. Green arrows indicate palmitoylation, activation, cystine import, or protective signaling, whereas red arrows or dashed red arrows indicate ROS/lipid peroxide accumulation, impaired palmitoylation, or ferroptosis-promoting consequences.

Ferroptosis is a non-apoptotic form of cell death driven by iron accumulation and dysregulated lipid peroxidation, characterized by peroxidation of polyunsaturated phospholipids, glutathione depletion, and failure of antioxidant defense systems [53]. Recent studies indicate that palmitoylation regulates ferroptosis primarily by modulating the stability and membrane-associated function of antioxidant proteins, cystine transporters, and lipid metabolism-related enzymes, thereby influencing cellular sensitivity to ferroptotic stimuli (Figure 5). GPX4 is a central suppressor of ferroptosis, functioning to detoxify membrane lipid peroxides and maintain redox homeostasis. Palmitoylation of GPX4 mediated by zDHHC8 or zDHHC20, and reversed by APT2, supports GPX4 stability and anti-ferroptotic function, thereby enhancing cellular resistance to lipid peroxidation [17,20]. Conversely, reduced GPX4 palmitoylation impairs its membrane localization and antioxidant function, leading to accumulation of lipid peroxidation products and increased susceptibility to ferroptosis [54]. Thus, palmitoylation not only affects GPX4 expression but also critically determines its functional capacity to buffer membrane lipid peroxidative damage.

In addition to GPX4, the key subunit of the system x_c^-^ transporter, SLC7A11, is also regulated by palmitoylation. zDHHC8-mediated palmitoylation of SLC7A11 supports its function in cystine uptake and glutathione synthesis, thereby facilitating ferroptosis resistance in glioblastoma models [27]. Conversely, increased depalmitoylation or impaired palmitoylation accelerates SLC7A11 degradation, weakens the intracellular redox environment, and increases susceptibility to ferroptosis [55]. Moreover, palmitoylation can regulate lipid metabolism–related proteins such as fatty acid synthase (FASN), thereby influencing membrane lipid composition and oxidative sensitivity. For example, palmitoylation of FASN enhances its catalytic activity, promoting fatty acid synthesis and membrane lipid remodeling, which supports membrane homeostasis under specific conditions [56]. Thus, the role of palmitoylation in ferroptosis is not unidirectional; rather, it modulates cellular tolerance to lipid peroxidative damage by regulating GPX4 antioxidant function, SLC7A11 membrane stability, and lipid metabolic reprogramming, thereby acting as a key molecular link between metabolic dysregulation and cell death.

### 4.5. Palmitoylation- and Autophagy-Related Cell Death

**Figure 6:**
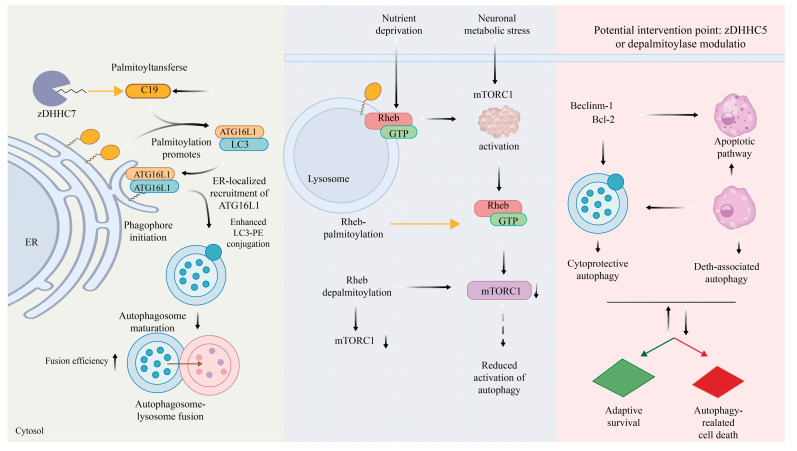
Palmitoylation regulates autophagy-related cell death by controlling autophagosome initiation, LC3 lipidation, autophagic flux, and nutrient-sensing pathways. zDHHC5-mediated palmitoylation of Beclin1 at Cys137 supports ATG14L-containing PI3KC3-C1 complex formation and lipid kinase activity, whereas reduced Beclin1 palmitoylation contributes to aging-associated autophagy decline. zDHHC7-mediated palmitoylation of ATG16L1 at Cys153 promotes its interaction with WIPI2B/RAB33B, LC3 lipidation, and autophagosome formation. Palmitoylation also influences autophagic flux, mTORC1-related nutrient sensing, and Beclin1–Bcl-2-associated crosstalk, thereby modulating the balance between protective autophagy and excessive autophagy-associated cell death under stress conditions. Yellow/orange elements indicate palmitoylation-related proteins or events, blue elements indicate autophagy-related structures or factors, purple elements indicate mTORC1 signaling, green arrows or diamonds indicate adaptive or cytoprotective outcomes, and red arrows or diamonds indicate apoptosis- or autophagy-related cell death. Solid arrows indicate activation, recruitment, fusion, or pathway progression, whereas gray or dashed arrows indicate regulatory or inhibitory processes.

Autophagy is a key process for maintaining cellular homeostasis, adapting to nutrient deprivation, and removing damaged organelles; however, under sustained or excessive activation, it can shift toward promoting cell death, known as autophagy-related cell death [57]. Recent studies indicate that palmitoylation participates in the dynamic balance of autophagy by regulating autophagosome initiation, membrane elongation, LC3 lipidation, autophagic flux, and nutrient-sensing pathways (Figure 6). Several autophagy-related gene (ATG) proteins have been identified as targets of palmitoylation. For example, zDHHC7-mediated palmitoylation of ATG16L1 at Cys153 facilitates its interaction with WIPI2B/RAB33B, promotes LC3 lipidation, and supports autophagosome formation [28]. Beclin1, a key component of the autophagy initiation complex, is also regulated by palmitoylation. Reduced zDHHC5-mediated palmitoylation of Beclin1 at Cys137 impairs ATG14L-containing PI3KC3-C1 formation and lipid kinase activity, thereby contributing to aging-associated autophagy decline [47,58]. These findings indicate that palmitoylation directly contributes to the initiation and maintenance of autophagy by modulating the membrane localization and complex assembly of core autophagy proteins.

In addition, palmitoylation regulates the transition between autophagy and cell death by modulating membrane dynamics and nutrient-sensing pathways. Upstream regulators such as Rheb depend on lipid modification for membrane localization and activity; enhanced palmitoylation sustains mTORC1 activity and suppresses autophagy, whereas reduced palmitoylation promotes autophagy activation. Under prolonged stress, this regulation may shift autophagy from a protective process toward a death-promoting one [12]. Palmitoylation may also contribute to crosstalk between autophagy and apoptosis by affecting the stability of the Beclin1–Bcl-2 complex [59]. Overall, palmitoylation regulates autophagy-related cell death primarily by controlling the membrane localization of core factors such as ATG proteins and Beclin1, vesicle maturation, and energy-sensing signaling, thereby serving as a key determinant of cell fate decisions between survival and death.

### 4.6. Palmitoylation-Mediated Crosstalk Among Cell Death Pathways

**Figure 7:**
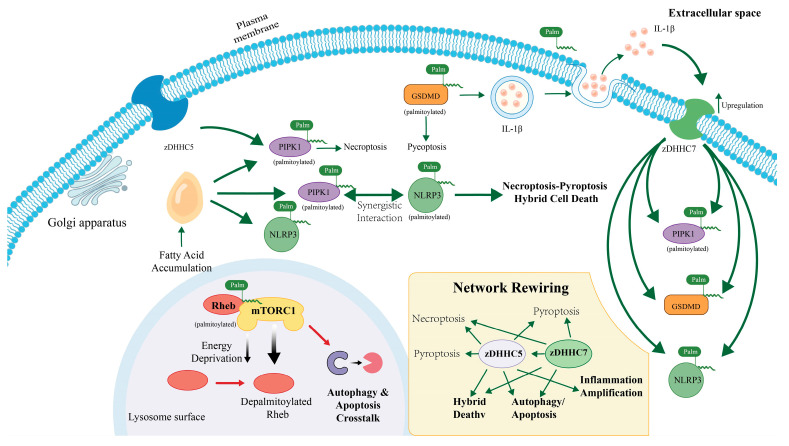
Palmitoylation acts as an integrated signaling hub coordinating crosstalk among multiple regulated cell death pathways. Palmitoylation regulates pathway-specific substrates involved in apoptosis, necroptosis, pyroptosis, ferroptosis, and autophagy-related cell death, while also acting on shared signaling nodes that connect these pathways. By controlling membrane localization, lipid raft organization, signaling complex assembly, metabolic adaptation, and inflammatory amplification, palmitoylation may influence the threshold, intensity, and interconversion of distinct cell death programs. Solid arrows indicate experimentally supported regulatory relationships, whereas dashed arrows indicate proposed or context-dependent crosstalk. This network-level regulation links lipid metabolism, membrane dynamics, inflammatory signaling, and cell fate determination. Green “Palm” labels and wavy lines indicate palmitoylation modification, green arrows indicate palmitoylation-related signaling or pathway activation, red arrows indicate inhibitory or attenuating effects, and black/bold arrows indicate downstream amplification or pathway crosstalk. Colored protein symbols represent pathway-associated molecules, including zDHHC enzymes, PIPK1, NLRP3, GSDMD, Rheb, and mTORC1, as indicated in the figure.

Although apoptosis, necroptosis, pyroptosis, ferroptosis, and autophagy-related cell death have distinct molecular execution mechanisms, they are not isolated processes but form highly interconnected regulatory networks under conditions such as stress, inflammation, and metabolic imbalance. Increasing evidence indicates that palmitoylation does not act on a single molecule within an individual pathway; rather, it serves as a key signaling hub linking different cell death modalities by regulating membrane localization, complex assembly, metabolic adaptation, and inflammatory amplification (Figure 7).

A key basis for crosstalk among different cell death pathways is the sharing of critical signaling molecules and reliance on similar membrane-associated regulatory events [60]. Palmitoylation participates in the cooperative or competitive regulation of multiple death pathways by targeting these shared nodes. For example, zDHHC5 not only mediates palmitoylation of pyroptosis-related proteins such as GSDMD but is also involved in the membrane-associated regulation of necroptosis factors like RIPK1, suggesting that a single palmitoyl acyltransferase may function as a hub across various forms of inflammatory cell death [61]. Similarly, proteins such as GPX4, SLC7A11, and Beclin1, which belong to ferroptosis and autophagy-related networks, may also be functionally interconnected through palmitoylation. Meanwhile, palmitoylation is closely linked to lipid metabolic status. In distinct experimental contexts, altered lipid availability or inflammatory stimulation has been reported to influence the palmitoylation of individual cell death-related proteins, such as RIPK1, NLRP3, GSDMD, and ferroptosis- or metabolism-associated regulators. However, whether these palmitoylation events are coordinately induced by elevated intracellular palmitate within the same pathological setting remains to be established. In contrast, under nutrient deprivation or energy stress, depalmitoylation of membrane-associated factors that sustain mTORC1 activity may enhance autophagy and further influence the thresholds of other cell death pathways [62].

Beyond metabolic coupling, palmitoylation also plays a broad role in amplifying inflammatory signaling and reshaping the immune microenvironment. For example, FASN-dependent MYD88 palmitoylation is required for TLR-mediated inflammatory signaling, supporting a mechanistic link between lipid metabolism, palmitoylation, and NF-κB-associated inflammatory activation [63]. Overall, the role of palmitoylation across multiple cell death modalities shares three common features: dependence on membrane localization and microdomain organization, regulation of complex assembly and signaling thresholds, and integration of metabolism, inflammation, and cell fate. These properties elevate palmitoylation from a single modification event to an integrative regulatory layer within cell death networks. In summary, palmitoylation-mediated signaling crosstalk indicates that cell death is not a linear process driven by a single pathway, but rather a networked output shaped by membrane-associated signaling, metabolic states, and inflammatory inputs; systematic analysis of this network will facilitate a deeper understanding and intervention of disease-related cell death. Together, these findings indicate that palmitoylation regulates cell death not only through pathway-specific substrates but also through shared signaling nodes across multiple death programs (Table 1).

**Table 1:** Representative palmitoylation-regulated mechanisms involved in regulated cell death pathways.

Cell Death Pathway	Palmitoylated Protein/Substrate	zDHHC Enzyme or Depalmitoylating Regulator	Palmitoylation Site	Functional Consequence	Experimental Model/Species	Evidence Level	Disease Relevance	Reference
Apoptosis	Fas/CD95	zDHHC7	Cys199	Promotes Fas stability, lipid raft localization, and Fas-mediated apoptotic signaling	Human colorectal cancer cells	Direct evidence	Immune regulation; cancer apoptosis resistance	[29]
Apoptosis	Bax	zDHHC3/zDHHC7/zDHHC11/zDHHC12/zDHHC21	Cys126	Promotes mitochondrial targeting, oligomerization, caspase activation, and intrinsic apoptosis	HEK293 cells, Cos7 cells, mouse tissues, Hodgkin B-cell lines	Direct evidence	Stress-induced apoptosis; cancer cell apoptosis resistance	[38]
Necroptosis	RIPK1	zDHHC5	Not fully defined	Licenses RIPK1 kinase activity and promotes downstream inflammatory cell death signaling	TNF-stimulated mammalian cell models; mouse inflammatory disease model	Direct evidence	Inflammatory diseases; tissue injury	[44]
Necroptosis	MLKL	Not fully defined	Not fully defined	Acylation/palmitoylation is associated with MLKL function during necroptotic execution	Mammalian cell models	Partial evidence	Inflammatory cell death	[14]
Pyroptosis	NLRP3	zDHHC5/ABHD17A	Cys837/Cys838	Promotes NLRP3–NEK7 interaction, inflammasome assembly, and activation	Macrophages and mouse inflammatory models	Direct evidence	NLRP3-driven inflammation; metabolic and chronic inflammatory diseases	[16]
Pyroptosis	GSDMD	zDHHC5/zDHHC9	Cys191 (human)/Cys192 (mouse)	Promotes GSDMD palmitoylation, oligomerization, membrane pore formation, and pyroptotic execution	THP-1 macrophages, iBMDMs, primary macrophages, and mouse inflammatory models	Direct evidence	Inflammasome activation and inflammatory diseases	[51]
Ferroptosis	GPX4	zDHHC8; zDHHC20; APT2	Cys66 reported	Stabilizes GPX4 and suppresses lipid peroxidation, thereby promoting ferroptosis resistance	Cancer cell models; tumor-bearing mouse models	Direct evidence	Cancer ferroptosis resistance; antitumor immunity	[17]
Ferroptosis	SLC7A11	zDHHC8	Not fully defined	Promotes SLC7A11 palmitoylation and supports cystine uptake, glutathione synthesis, and ferroptosis resistance	Glioblastoma cell and tumor models	Direct evidence	Glioblastoma; ferroptosis resistance	[27]
Autophagy-related cell death	ATG16L1	zDHHC7	Cys153	Promotes ATG16L1–WIPI2B/RAB33B complex formation, LC3 lipidation, and autophagosome formation	*ATG16L1*-KO HeLa cells; mammalian autophagy models	Direct evidence	Autophagy dysfunction	[28]
Autophagy-related cell death	Beclin1	zDHHC5	Cys137	Promotes ATG14L-containing PI3KC3-C1 formation, lipid kinase activity, and autophagic flux	Mammalian cell stress models	Direct evidence	Aging, stress responses	[56]

## 5. Palmitoylation and Disease Mechanisms: From Cell Death Dysregulation to Pathological Remodeling

**Figure 8:**
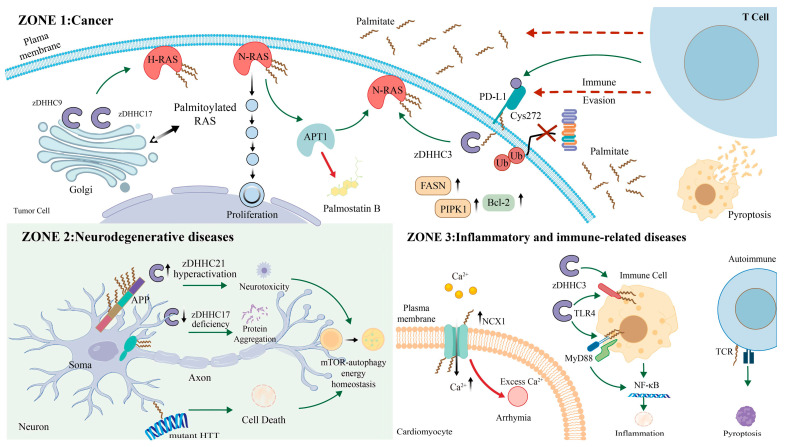
Pathological relevance and therapeutic targeting of protein palmitoylation in disease-associated cell death remodeling. Dysregulated palmitoylation contributes to disease progression in cancer, neurodegenerative diseases, and inflammatory or immune-related diseases by reshaping protein localization, signaling complex assembly, cell death sensitivity, and inflammatory outputs. In cancer, palmitoylation may promote oncogenic signaling, immune evasion, and ferroptosis resistance. In neurodegenerative diseases, altered palmitoylation may impair synaptic function, protein trafficking, autophagy, and neuronal survival. In inflammatory and immune-related diseases, palmitoylation regulates inflammasome activation, gasdermin-mediated pyroptosis, necroptotic signaling, and inflammatory amplification. Targeting zDHHC enzymes, depalmitoylating enzymes, or specific palmitoylated substrates may provide potential therapeutic strategies for restoring cell death balance and limiting pathological remodeling. The three zones indicate cancer, neurodegenerative diseases, and inflammatory or immune-related diseases, respectively. Purple symbols indicate zDHHC enzymes, brown wavy lines indicate palmitate modification, green arrows indicate palmitoylation-related activation or signaling enhancement, red arrows indicate inhibitory or detrimental effects, and red dashed arrows indicate immune-evasion or negative regulatory routes.

Dysregulation of cell death is a fundamental driver of the development and progression of many diseases, and palmitoylation—by linking membrane signaling, metabolic status, and cell fate determination—has emerged as a key regulatory mechanism for understanding pathological remodeling [64]. Under pathological conditions, aberrant palmitoylation does not merely alter the localization and/or stability of individual proteins; rather, it may reshape disease-related signaling networks by affecting multiple cell death pathways, inflammatory amplification, immune evasion, and metabolic reprogramming (Figure 8). Importantly, the pathological consequence of palmitoylation is highly context-dependent. In some cases, palmitoylation events may act as disease-driving mechanisms by stabilizing oncogenic, pro-inflammatory, or cell death-executing proteins; in other settings, altered palmitoylation may represent an adaptive or secondary response to metabolic stress, oxidative injury, chronic inflammation, or therapeutic pressure. Therefore, palmitoylation should be interpreted not simply as a universal pro-death or anti-death modification, but as a disease-context-dependent regulatory layer that determines the intensity, timing, and interconversion of cell death modalities in specific tissues and pathological microenvironments. Representative examples linking dysregulated palmitoylation to disease-related signaling and pathological remodeling are summarized in Table 2.

### 5.1. Palmitoylation and Tumorigenesis and Progression

Sustained tumor growth relies on enhanced proliferative signaling, suppression of cell death, metabolic reprogramming, and immune evasion, all of which can be influenced by palmitoylation. In cancer cells, palmitoylation may function as a disease-driving mechanism when it stabilizes oncogenic proteins or strengthens pro-survival signaling. For example, the oncogenic activity of RAS critically depends on its plasma membrane localization, a process requiring palmitoylation; aberrant expression of palmitoyl acyltransferases such as zDHHC9 and zDHHC17 promotes RAS cycling between the Golgi and plasma membrane, thereby sustaining MAPK/ERK signaling and enhancing tumor cell proliferation and survival [39]. Inhibition of the depalmitoylating enzyme APT1 by agents such as palmostatin B disrupts this process and induces cell death [65]. In addition, zDHHC3-mediated palmitoylation of PD-L1 at Cys272 reduces its ubiquitination and lysosomal degradation, prolonging its membrane residence and enhancing suppression of T cell activity [30,66]. Recent studies further indicate that palmitoylation of ferroptosis-related regulators, including GPX4 and SLC7A11, can support antioxidant defense, cystine uptake, and ferroptosis resistance in cancer cells, thereby contributing to therapy tolerance in specific tumor contexts [17,20,27,67]. However, not all palmitoylation alterations in tumors should be interpreted as primary oncogenic drivers; some may represent adaptive or secondary responses to hypoxia, nutrient stress, inflammatory signals, or anticancer therapy [68]. Therefore, in tumorigenesis and progression, palmitoylation should be viewed as a context-dependent regulatory layer that can support oncogenic signaling, immune evasion, and cell death resistance, while its causal contribution needs to be evaluated according to substrate identity, modification site, enzyme specificity, and experimental evidence.

### 5.2. Palmitoylation and Neurodegenerative Diseases

In the nervous system, palmitoylation is widely involved in synaptic transmission, receptor trafficking, axonal transport, and membrane protein homeostasis, and its dysregulation is closely associated with various neurodegenerative diseases. Unlike in cancer, where palmitoylation often supports aberrant cell survival, dysregulated palmitoylation in neurodegeneration more commonly impairs synaptic stability, protein quality control, and organelle homeostasis, thereby increasing neuronal vulnerability to cell death [50]. Key synaptic proteins such as PSD-95, SNAP25, and GluA2 depend on palmitoylation for proper membrane localization and synaptic function; disruption of this modification impairs synaptic transmission and neuronal plasticity [69]. In Alzheimer’s disease, altered palmitoylation of BACE1 and zDHHC21/Fyn-related signaling has been associated with amyloid production, Tau-related pathology, and synaptic dysfunction. In Huntington’s disease, impaired HIP14/zDHHC17-related palmitoylation of huntingtin and huntingtin-associated synaptic substrates disrupts neuronal trafficking, synaptic homeostasis, and neuronal signaling, thereby contributing to neuronal vulnerability [70,71,72,73]. In addition, palmitoylation regulates the mTOR–autophagy axis and lysosome-related protein turnover, influencing the clearance of abnormal proteins and damaged organelles; disruption of this process may increase metabolic stress and sensitize neurons to cell death [74]. Therefore, in neurodegenerative diseases, palmitoylation dysregulation should be viewed less as a simple on/off trigger of a single death pathway and more as a progressive disturbance of synaptic organization, proteostasis, and organelle quality control. Depending on the disease stage and affected substrate, such changes may act either as early contributors to neuronal dysfunction or as secondary amplifiers of ongoing neurodegenerative injury.

### 5.3. Palmitoylation and Inflammatory and Immune-Related Diseases

In inflammatory and immune-related diseases, palmitoylation plays a particularly prominent role because inflammasome activation, inflammatory cell death, and cytokine release all depend on the precise organization of membrane-associated signaling molecules. As a dynamic and reversible lipid modification, palmitoylation regulates these membrane-proximal signaling nodes, thereby contributing to immune homeostasis and inflammatory disease progression [75]. For example, palmitoylation of adaptor proteins such as MyD88 enhances their membrane localization and downstream NF-κB signaling, promoting the expression and release of pro-inflammatory cytokines [24]. Similarly, palmitoylation of inflammation-related molecules such as STAT3 and GRK6 is closely associated with macrophage activation, remodeling of the inflammatory microenvironment, and the intensity of immune responses [36]. In addition, as discussed above, palmitoylation regulates zDHHC5–NLRP3–NEK7 inflammasome assembly and zDHHC5/zDHHC9-mediated GSDMD pore formation, thereby forming a potential “membrane localization–inflammasome activation–cell lysis–cytokine release” amplification loop. Therefore, in infection, metabolic inflammation, and autoimmune diseases, dysregulated palmitoylation may simultaneously amplify pyroptosis, necroptosis, and associated inflammatory outputs, influencing both the duration of inflammation and the extent of tissue damage. Meanwhile, T cell receptor signaling, macrophage polarization, and cytokine release also depend on membrane microdomain organization and signaling complex stability [4]. Thus, palmitoylation contributes to inflammatory and immune-related diseases not only by regulating individual immune signaling proteins but also by coordinating innate immune activation, inflammatory cell death, and immune-cell functional remodeling. However, whether a specific palmitoylation event acts as an initiating driver or a secondary amplifier of inflammation should be evaluated according to the disease context, target substrate, and available functional evidence.

### 5.4. Context-Dependent Remodeling of Disease Signaling Networks

With advances in lipidomics, proteomics, and spatial omics technologies, palmitoylation is no longer regarded as a local modification at the single-protein level, but is increasingly understood as a regulatory mechanism with network-organizing capacity [76]. In various disease contexts, aberrant palmitoylation is not confined to a single substrate or pathway; instead, it may involve coordinated alterations in multiple membrane-associated molecules, metabolic regulators, immune signaling proteins, and cell death nodes, thereby contributing to global remodeling of disease-related signaling networks [77].

From a systems perspective, the pathological outcome of palmitoylation-regulated cell death can be interpreted through three interconnected determinants. First, substrate identity largely determines the direction of regulation: palmitoylation of pro-death molecules such as Fas, Bax, RIPK1, MLKL, NLRP3, or GSDMD may enhance death signaling, whereas palmitoylation of pro-survival or stress-adaptive proteins such as Bcl-2, Akt, PD-L1, SLC7A11, or GPX4 may suppress cell death or promote immune evasion and stress resistance [78]. Second, disease microenvironment determines the functional consequence of the same modification. In tumors, palmitoylation frequently supports oncogenic signaling, immune escape, and resistance to apoptosis or ferroptosis; in neurodegenerative diseases, altered palmitoylation more often disrupts synaptic stability, lysosomal clearance, and neuronal proteostasis; in inflammatory diseases, palmitoylation tends to amplify inflammasome activation, necroptosis, pyroptosis, and cytokine release [79]. Third, the interpretation of palmitoylation events should depend on the strength of available evidence. Palmitoylation events supported by clearly identified modification sites, specific zDHHC or depalmitoylating enzymes, and functional validation experiments are more likely to represent direct regulatory mechanisms in disease progression. In contrast, changes observed only at the expression or correlation level should be interpreted more cautiously, as they may reflect secondary responses to disease-associated stress or inflammation rather than primary pathogenic drivers [80,81].

At the network level, palmitoylation can influence disease progression through several convergent routes: a single palmitoyl acyltransferase may target multiple functionally related substrates across different pathways, thereby linking cell death, inflammation, and metabolic adaptation; palmitoylation is sensitive to cellular metabolic status and is often coupled to lipid metabolic reprogramming, oxidative stress, and inflammatory inputs; and palmitoylation-dependent changes can amplify pathological effects at the tissue and organ levels by modulating immune cell function, inflammatory mediator release, and the local microenvironment [69,82,83]. Therefore, palmitoylation is better understood as a multi-layer regulatory node connecting “lipid metabolism–membrane signaling–cell death–disease phenotypes.” Integrating multi-omics analyses with network modeling and artificial intelligence approaches may facilitate construction of three-dimensional regulatory networks of palmitoylation, helping to distinguish driver events from adaptive or passenger changes and enabling more precise intervention strategies. These examples suggest that palmitoylation acts as a cross-disease regulatory layer that integrates lipid metabolism, membrane signaling, inflammatory cell death, immune responses, and tissue remodeling (Table 2).

Collectively, current evidence suggests that the biological outcome of palmitoylation depends on multiple context-dependent determinants rather than a single unifying mechanism. First, substrate identity appears to be a major factor: palmitoylation of pro-death regulators such as Fas, RIPK1, NLRP3, and GSDMD generally enhances death signaling, whereas palmitoylation of stress-adaptive or pro-survival proteins such as PD-L1, GPX4, and SLC7A11 may suppress cell death or promote stress resistance. Second, metabolic and inflammatory conditions may reshape palmitoylation-dependent signaling by altering lipid availability, membrane organization, and enzyme activity. Third, the same palmitoylation event may produce distinct outcomes depending on cell type, organelle localization, and disease stage. Therefore, palmitoylation should be viewed as a context-dependent signaling regulator rather than a uniformly pro-death or anti-death modification [20,27,30,84]. From a therapeutic perspective, commonly used compounds such as 2-bromopalmitate, palmostatin B, ML348, and ML349 remain primarily experimental tool compounds. 2-Bromopalmitate broadly inhibits palmitoylation and may cause off-target metabolic interference and cytotoxicity, whereas palmostatin B, ML348, and ML349 are mainly used to modulate depalmitoylating enzymes and still require further evaluation of specificity, bioavailability, and in vivo safety [85,86,87].

**Table 2:** Disease-associated remodeling of palmitoylation-regulated cell death networks.

Disease Category	Disease/Type	Key Regulator or Substrate	Dominant RCD Phenotype	Mechanistic Role	Pathological Consequence	Potential Pathological Role	Reference
Cancer	RAS-driven cancers (pancreatic cancer, colorectal cancer, melanoma and others)	zDHHC9; zDHHC17; RAS	Apoptosis resistance/pro-survival signaling	Palmitoylation promotes RAS cycling between the Golgi apparatus and plasma membrane, sustaining MAPK/ERK signaling and oncogenic activity.	Enhanced tumor cell proliferation, survival, and metabolic adaptation.	Likely driver	[39]
Cancer	Breast cancer/endocrine-resistant breast cancer	zDHHC22, mTOR	Apoptosis resistance/pro-survival signaling	zDHHC22-mediated mTOR palmitoylation modulates AKT signaling and endocrine therapy response.	Endocrine therapy resistance and tumor progression.	Context-dependent	[66]
Cancer	Melanoma, lung cancer and other tumors	zDHHC3, PD-L1	Immune evasion; resistance to immune-mediated killing	PD-L1 Cys272 palmitoylation prevents ubiquitination-dependent degradation.	Prolonged PD-L1 membrane retention suppresses T-cell-mediated tumor killing.	Likely driver	[30]
Cancer	Glioblastoma and therapy-resistant tumors	zDHHC8; zDHHC20; SLC7A11; GPX4	Ferroptosis resistance	Palmitoylation stabilizes ferroptosis-suppressive proteins and supports cystine uptake or antioxidant defense.	Tumor cells acquire resistance to lipid peroxidation and ferroptotic death.	Likely driver/context-dependent	[17,24,27]
Neurodegenerative diseases	Alzheimer’s disease	zDHHC21; Fyn-related regulators; BACE1	Neuronal injury; autophagy-related dysfunction	Palmitoylation promotes lipid raft enrichment of BACE1 and may enhance Fyn-related signaling associated with Tau pathology.	Increased Aβ production, Tau phosphorylation, synaptic dysfunction and cognitive decline.	Context-dependent/secondary amplifier	[70,71]
Neurodegenerative diseases	Huntington’s disease	zDHHC17/HIP14; huntingtin	Neuronal dysfunction; apoptosis susceptibility	Impaired palmitoylation of synaptic proteins and huntingtin-associated substrates disrupts neuronal signaling and trafficking.	Synaptic instability, impaired axonal transport and reduced neuronal survival.	Likely contributor	[72,73]
Inflammatory and immune-related diseases	NLRP3-driven inflammatory diseases; metabolic inflammation	NLRP3; zDHHC5; ABHD17A	Pyroptosis amplification	zDHHC5-mediated NLRP3 palmitoylation promotes NLRP3–NEK7 interaction and inflammasome activation, whereas ABHD17A reverses this modification.	Enhanced IL-1β/IL-18 release, inflammatory cell death and tissue injury.	Likely driver/amplifier	[16]
Inflammatory and immune-related diseases	Inflammasome-associated inflammatory diseases	GSDMD; zDHHC5; zDHHC9	Pyroptosis execution	ROS-dependent GSDMD palmitoylation promotes oligomerization, membrane pore formation and pyroptotic execution.	Cell lysis, cytokine release and inflammatory amplification.	Likely driver/amplifier	[51]

## 6. Conclusions and Future Directions

Palmitoylation, as a dynamic and reversible lipid post-translational modification, has emerged as a key molecular mechanism regulating cell fate. It not only governs protein membrane localization and complex assembly but also participates in the initiation, amplification, and termination of cell death signaling. Multiple pathways—including apoptosis, necroptosis, pyroptosis, ferroptosis, and autophagy-related cell death—depend, to varying extents, on the spatiotemporal dynamics of palmitoylation. At the disease level, accumulating evidence links aberrant palmitoylation to tumorigenesis, neurodegenerative disorders, and inflammatory and immune dysregulation. The dynamic and reversible modification system composed of zDHHC family palmitoyl acyltransferases and depalmitoylating enzymes such as APTs and PPTs provides a highly plastic molecular basis for cellular signaling; disruption of this balance often leads to dysregulated cell death and disease progression.

From a therapeutic perspective, palmitoylation-regulated cell death may be targeted at multiple levels, including zDHHC enzymes, depalmitoylating enzymes, and specific palmitoylated substrates. However, these strategies remain emerging and context-dependent rather than clinically established therapeutic approaches, because enzyme selectivity, substrate overlap, tissue specificity, bioavailability, and in vivo safety remain major challenges.

Several limitations should also be recognized. The strength of evidence varies substantially among different palmitoylation events; some are supported by clearly identified modification sites, specific enzymes, and functional validation experiments, whereas others remain based mainly on association or prediction. Moreover, zDHHC enzymes and depalmitoylating enzymes often exhibit substrate overlap and context-dependent activity, making it difficult to assign a single enzyme to a single substrate or pathway. In addition, quantitative information on palmitoylation turnover, enzyme-specific kinetics, and substrate half-lives remains limited, which restricts direct comparison with faster and better-characterized phosphorylation–dephosphorylation cycles. Future studies should therefore combine site-specific validation, spatial proteomics, lipidomics, disease-relevant models, and network-based analyses to distinguish causal regulatory mechanisms from secondary adaptive changes. Integrating multi-omics approaches with artificial intelligence-assisted modeling may further facilitate construction of systems-level regulatory networks linking palmitoylation, regulated cell death, and disease progression.

In summary, palmitoylation, as a critical molecular hub linking lipid metabolism and cell fate determination, is emerging as a frontier topic in cell death research and disease biology. A deeper understanding of its dynamic regulation and network-level functions will not only advance our knowledge of programmed cell death but also provide new avenues for the prevention and treatment of complex diseases.

## Data Availability

No new data were created or analyzed in this study. Data sharing is not applicable to this article.

## Supplementary Materials

The following supporting information can be downloaded at: https://www.mdpi.com/article/10.3390/biom16060853/s1, Figure S1. PRISMA-style flow diagram of literature identification, screening, eligibility assessment, and study inclusion.

## Abbreviations

The following abbreviations are used in this manuscript:

RIPK1	receptor-interacting protein kinase 1
GSDMD	gasdermin D
GPX4	glutathione peroxidase 4
zDHHC	zinc finger Asp-His-His-Cys
PATs	palmitoyl acyltransferases
PPT1	palmitoyl protein thioesterase 1
NCL	neuronal ceroid lipofuscinosis
LAT	linker for activation of T cells
PCD	programmed cell death
FADD	fas-associated death domain protein
DISC	death-inducing signaling complex
TNFR	tumor necrosis factor receptor
TRAIL-R	TNF-related apoptosis-inducing ligand receptor
MLKL	mixed lineage kinase domain-like
DAMPs	damage-associated molecular patterns
NLRP3	NLR family pyrin domain-containing 3
MAP1LC3	microtubule-associated protein 1 light chain 3
