# Supplementary material for: Protein Palmitoylation as a Molecular Switch Linking Regulated Cell Death and Disease
Source: Biomolecules. 2026 Jun 11;16(6):853. doi: 10.3390/biom16060853 (PMC13296868; doi:10.3390/biom16060853)
Supplement: Supplementary file 1 [file biomolecules-16-00853-s001.zip › biomolecules-4310138-supplementary.pdf]

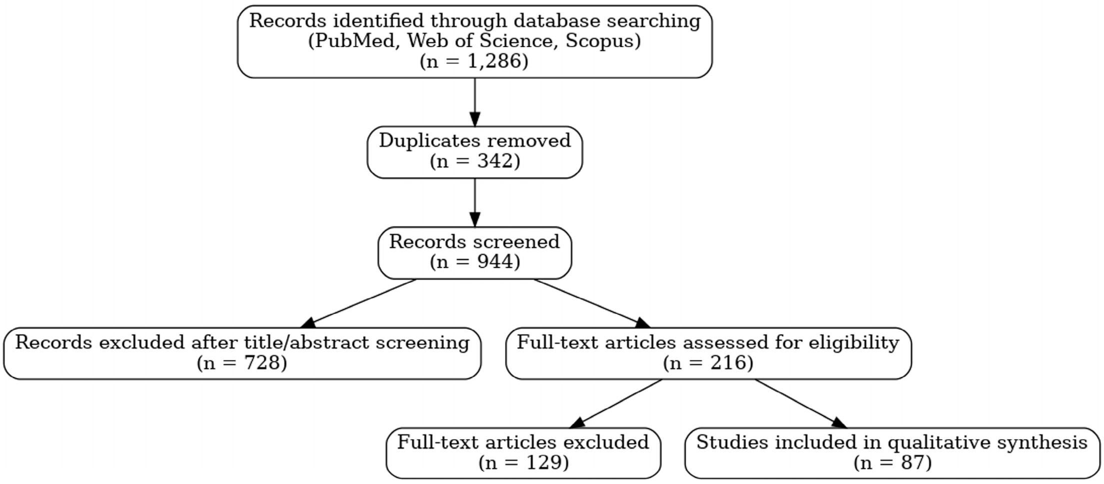

Figure S1. PRISMA-style flow diagram of literature identification, screening, eligibility assessment, and study inclusion. Literature records were identified from PubMed, Web of Science, and Scopus databases. After duplicate removal, records were screened based on titles and abstracts, followed by full-text eligibility assessment. Studies meeting the predefined inclusion criteria were incorporated into the qualitative synthesis of this review.
